# Risk factors on testicular function in adolescents

**DOI:** 10.1007/s40618-022-01769-8

**Published:** 2022-03-14

**Authors:** F. Cargnelutti, A. Di Nisio, F. Pallotti, M. Spaziani, M. G. Tarsitano, D. Paoli, C. Foresta, Talent Group

**Affiliations:** 1 Laboratory of Seminology-Sperm Bank “Loredana Gandini”, Department of Experimental Medicine, “Sapienza” University of Rome, Viale del Policlinico 155, 00161 Rome, Italy; 2 Department of Medicine, Operative Unit of Andrology and Medicine of Human Reproduction, University of Padova, Via Giustiniani, 2, 35128 Padua, Italy; 3 Department of Experimental Medicine, “Sapienza” University of Rome, Viale del Policlinico 155, 00161 Rome, Italy

**Keywords:** Puberty, Varicocele, Cancer, Diabetes, Cryptorchidism, Obesity

## Abstract

**Purpose:**

Adolescence represents an important window for gonadal development. The aim of this review is to carry out a critical excursus of the most recent literature on endogenous and exogenous risk factors related to testicular function, focusing the research on adolescence period.

**Methods:**

A comprehensive literature search within PubMed was performed to provide a summary of currently available evidence regarding the impact on adolescence of varicocele, cryptorchidism, cancer, diabetes, lifestyle factors, endocrine disruptors, obesity and sexually transmitted diseases. We focused on human studies that evaluated a possible impact of these factors on puberty timing and their effects on andrological health.

**Results:**

Evidence collected seems to suggest that andrological health in adolescence may be impaired by several factors, as varicocele, cryptorchidism, and childhood cancer. Despite an early diagnosis and treatment, many adolescents might still have symptoms and sign of a testicular dysfunction in their adult life and at the current time it is not possible to predict which of them will experience andrological problems. Lifestyle factors might have a role in these discrepancies. Most studies point out towards a correlation between obesity, insulin resistance, alcohol, smoking, use of illegal drugs and testicular function in pubertal boys. Also, endocrine disruptors and sexually transmitted diseases might contribute to impair reproductive health, but more studies in adolescents are needed.

**Conclusion:**

According to currently available evidence, there is an emerging global adverse trend of high-risk and unhealthy behaviors in male adolescents. A significant proportion of young men with unsuspected and undiagnosed andrological disorders engage in behaviors that could impair testicular development and function, with an increased risk for later male infertility and/or hypogonadism during the adult life. Therefore, adolescence should be considered a key time for intervention and prevention of later andrological diseases.

## Introduction

As is well known, the testicle performs two main functions, closely integrated with each other although taking place in two separate compartments: androgens synthesis and spermatogenesis. The maintenance of these two important functions is closely linked to the integrity of the hypothalamus-pituitary-gonad (HPG) axis, therefore any insult that could affect one of the three components of the axis may result in a dysfunction in the production of androgens and/or spermatozoa. There are several conditions that may cause testicular dysfunction, which can be divided into two large groups: endogenous, linked to intrinsic risk factors, and exogenous causes, associated to external and strictly lifestyle-dependent risk factors. Among the most important endogenous causes, varicocele, cryptorchidism, cancer and type I diabetes deserve particular mention for their potential negative influence on gonadic function [1–4], while among the exogenous factors, life style, endocrine disruptors, obesity, type II diabetes (DMT2) and sexually transmitted diseases should be carefully considered, as they represent emerging risk factors for testicular failure [5–7]. In addition to the type of insult, the moment of life in which it happens may be crucial in the manifestation of testicular damage. Adolescence represents exactly a critical window, since it is a period of important physiological changes that requires a perfect hormonal balance. In view of the above, the purpose of this review is to carry out a critical excursus of the most recent literature on endogenous and exogenous risk factors related to testicular function, focusing the research on adolescence period.

## Endogenous causes

### Effects of varicocele

Varicocele is believed to cause infertility by disrupting spermatogenesis. It has been repeatedly reported an association between impaired semen parameters and varicocele, but exact pathological mechanisms are still debated, possibly including hypoxia and increased oxidative stress and increased scrotal temperature, among others [8]. In young adults, semen parameters may be worse than controls but still within WHO reference limits [8]. In adolescents, it seems a rather common condition, as its prevalence ranges between 7.8 and 14.1%, in boys aged 11–14 and 15–19 years, respectively [9]. Varicocele management in adolescents represents a controversial topic since studies in the pediatric and adolescent population are limited. Many studies evaluated only testicular volume, probably because of the difficulty in proposing semen analysis to adolescents [10]. Testicular volume is indeed considered a fairly reliable marker of spermatogenesis [11, 12], but it cannot replace semen analysis. Moreover, testicular growth is normal in pubertal development and asynchronous growth in adolescents with a varicocele may be transient, with a subsequent equalization [13], possibly depending on initial Tanner stage [14]. Several meta-analyses have been conducted on this topic. Nork et al. performed two meta-analysis in young boys aged 15–24 years, evaluating both the impact of varicocele and the effect of treatment on semen parameters. The authors included 10 studies in both hypothesis and concluded that varicocele in youth negatively affect sperm parameters, while the treatment results in a moderate improvement of sperm density and motility [15]. On the contrary, a successive meta-analysis by Zhou et al. considering seven studies reported only an improvement of adolescents’ testicular volumes after treatment, without any significant difference about sperm parameters [16]. Taking in consideration only randomized-controlled trials performed in children and adolescents up to 21 years old, Locke et al. concluded that there is low to moderate level of evidence that treatment of adolescent varicocele may improve testicular size/growth and sperm concentration [17]. Similar results were recently reported by Silay et al., who considered 98 articles (only 12 RCTs) including 16,130 patient aged 7–21 years [18]. Only few studies evaluated hormonal status in adolescents, with some of them reporting low inhibin B levels [19–22]. These findings suggest a possible testicular dysfunction, in particular of Sertoli cells, since inhibin B is secreted by these cells. Only two studies evaluated paternity rates after varicocele treatment in adolescence, with different results [23, 24]. A possible cause of infertility may be the elevated sperm DNA fragmentation, as high levels of this parameter have been associated with low fertility [25] and recurrent pregnancy loss [26]. Higher values had been observed in adolescents with varicocele, despite no alterations in semen parameters [27, 28]. In the same way, treatment may improve DNA fragmentation, though semen characteristics may remain the same [29].

In conclusion, evidence collected in children and adolescents seems to suggest a negative impact of varicocele on testicular function. However, available data are heterogeneous and this problem prevents the development of standardized guidelines about varicocele management in adolescents. Actually, current guidelines by European Association of Urology (EAU) do not provide a specific indication, affirming that adolescent varicocele is often overtreated [30]. On the contrary, American Society for Reproductive Medicine (ASRM), American Urological Association (AUA), Society for Male Reproduction and Urology (SMRU) suggest treatment of adolescent varicocele in the case of decreased testicular volume or sperm abnormalities [31]. Considering the paucity of literature and the lack of quality evidence, further studies are surely needed in order to identify who may possibly benefit from varicocele treatment in adolescence age.

### Effects of congenital cryptorchidism

Congenital undescended testis or cryptorchidism is one of the most common congenital malformations in boys. A prevalence at birth among boys with birth weight more than 2500 g of 1.8–8.4% has been reported. However, prevalence at the age of 3 months is 0.9–1.8%, due to spontaneous testicular descent [32]. Cryptorchidism is considered a classical risk factor of future gonadal dysfunction and increased incidence of testicular germ cell tumors. It does not seem to interfere with normal pubertal development, even if few data are available [33, 34]. Sadov et al. evaluated the onset of puberty in a longitudinal case–control study. Precise age at the onset of testicular growth was obtained for 95 boys (58 controls and 37 cases), with no significant difference (11.7 and 11.8 years in cases and controls, respectively), though subsequent testicular growth seemed to be impaired in cryptorchid boys [33]. In a large cohort study of 7698 boys (196 with cryptorchidism), Arendt et al. found no correlation between pubertal development and cryptorchidism. However, pubertal advance was evaluated through web-based questionnaires [34].

Many studies evaluated semen parameters in previously cryptorchid boys, with most reporting poorer sperm quality in this population [35–42]. Consequently, recent studies are focusing especially on the correct timing of surgical treatment, being late interventions theoretically associated with greater damage. Recently, in a large case–control study Rohayem et al. observed higher mean LH and FSH levels and lower mean testosterone levels, bi-testicular volumes and sperm concentration in previously cryptorchid men. Lowest mean sperm concentration was found in those with bilateral undescended testes. In addition, they found that age at correction (median: 6 years) was inversely correlated with testicular volumes and sperm concentration, and positively correlated with FSH and LH, but not with serum testosterone. Results suggest that correction of cryptorchidism should be performed early during infancy [35]. In the same way, in a large Australian population-based cohort study undescended testes was associated with a more than two times increase in risk of testicular cancer, with a 21% reduction in paternity, and with a two times increase in use of ART. Interestingly, the Authors calculated that for every 6 months delay in orchidopexy, there was a 6% increase in risk of testicular cancer, a 5% increase in risk of future use of ART, and a 1% reduction in paternity [43]. In 2001, Cortes et al. investigated 1335 cryptorchid boys with biopsy at surgery and they recorded an association between lack of germ cells, that appeared after 18-months of age with an increasing age-related frequency, and consequent risk of infertility [44]. Testicular histology at surgery might represent an important prognostic marker of subsequent normal spermatogenesis [42, 45]. Several studies reported a correlation between histology and age at surgery, with an age-related worsening of histological characteristics [46–50]. In a recent meta-analysis, the Authors reported that testicular volume was greater and there were more spermatogonia per tubule in infants undergoing early orchidopexy [51]. As a matter of fact, up to 25% of cryptorchid boys who underwent orchiopexy within the first year of life may have a reduced number of germ cells, being at risk of infertility despite early surgery [52]. In infants with unilateral cryptorchidism, similar histological alterations but less severe were found in the contralateral descended testes, suggesting that a deficiency in the hypothalamic-pituitary axis might lead to a failure to establish an adequate stem cell pool [48]. Considering all data literature, current guidelines recommend early orchiopexy, in the absence of spontaneous testicular descent by 6 months, before the age of 12 [53] or by 18 months at latest [54, 55].

In conclusion, although optimal timing of orchiopexy is not completely clarified, there is strong evidence suggesting that early surgery in cryptorchid boys is advantageous to reduce gonadal damage. However, up to 20–25% may still have fertility problems in their future. For this reason, a complete andrological evaluation of these boys should be always performed, during puberty and later.

### Effects of diabetes mellitus type-1

Diabetes mellitus type-1 (DMT1) is a chronic disease with a typical onset in childhood. This disease is characterized by the destruction of pancreatic β-cells, often by autoimmune processes, which generally results in an insulin deficit [56]. Complications may involve many apparatuses. The reproductive system, in particular, may be affected from DM at various levels: hypothalamic-pituitary–gonadal axis, spermatogenesis and ejaculation mechanism [57]. Because of its onset in childhood, DMT1 may affect pubertal development. In a recent cross-sectional study, Gaete et al. evaluated puberty timing in 148 DMT1 boys aged 7–19 years. The Authors observed that boys at the final stages of puberty (genital Tanner 4 and 5) and at genital Tanner stage 2 were younger than the control group, suggesting an earlier age of onset and an earlier age of final pubertal events in DMT1 boys [58]. The same group observed no significant differences in hormonal values of pubertal DMT1, though testosterone was significantly higher at the end of puberty in comparison with controls [59], as previously reported in both types of diabetics [60]. On the contrary, a large retrospective study showed a delay of pubertal onset (but not of sexual maturity) in DMT1 children, in comparison with general population. However, data were obtained by a national database, with numerous participating centers, and no control group was recruited [61]. Further data on adolescents are not available and little is known about DMT1 effects on reproductive health of older boy. Large retrospective epidemiological studies reported that both DMT1 women and men had a smaller number of live births than controls, but semen analysis was not performed [62, 63]. A negative effect on semen parameters had been reported by numerous studies on animal models [64–67]. Human clinical studies do not confirm these results, with several reporting altered semen parameters [68–75] and some observing no differences [76–81]. Many factors may cause these discrepancies. First, these studies have a very small caseload and different outcomes. Moreover, analyzed populations are widely heterogeneous for several characteristics, such as disease duration, the presence of diabetes complications and age, though aging seems to be an important factor in impairing spermatogenesis [82]. Finally, a complete andrological evaluation was not performed in most studies. Pergialiotis et al. [83] performed a meta-analysis to summarize, founding a correlation between DMT1, infertility and altered sperm parameters, though only sperm motility was significantly reduced. More recently, two Italian studies by the same group [74, 75] reported a significant reduction of progressive motility in patients with DMT1, with lower values when the duration of illness was longer than 10 years. The Authors also reported the reduction of mitochondrial membrane potential, an early event in the apoptosis process that can be the result of oxidative stress and may anticipate the subsequent decrease in sperm motility [74, 75].

Regarding hormonal function, most studies showed no alterations in DMT1 patients [69, 73–75], with only two reporting significant modifications [70, 71]. In Pergialiotis et al. meta-analysis [83], overall analysis showed no statistically significant differences. This result is probably influenced by insulin therapy, as insulin receptors are normally expressed on hypothalamus, olfactory bulb, and pituitary gland [84]. However, in a recent paper Maiorino et al. [85] observed a higher prevalence of erectile dysfunction in young DMT1 patients compared with controls, suggesting the important role of psychological factors in this age range, which should not be ignored. In conclusion, DMT1 is a rare disease that might have a deleterious effect on male reproductive health. For this reason, a complete andrological evaluation of these patients should never be neglected.

### Effects of childhood cancer

Prevalence of cancer in children and adolescents has increased over the last decades, though rates have stabilized over the latest years. Most commonly types of cancer in prepubertal boys and adolescents are leukemias, lymphomas, brain, and other central nervous system tumors, whereas testicular germ cell tumors are frequent in adolescents. At the same time, death rates in adolescents have declined continuously, with an overall reduction of 65% in comparison with 1970 [86]. Consequently, life expectancy for childhood cancer survivors has remarkably improved. New treatments are responsible of this improvement, but their gonadotoxic effects may lead to several complications. Pubertal disorders seem to affect almost exclusively brain tumors patients, because of hypothalamic-pituitary axis damage [87], whereas reproductive impairment is more common. Oncofertility is an emerging field that involves several fertility preservation strategies for patients diagnosed with cancer [88]. Prepubertal testis seems to be highly sensitive to gonadotoxic treatments [89]. Gonadotoxic effect of the different chemotherapeutic agents cannot be easily assessed, as it depends on many factors such as dosage and duration of treatment, age of patients and their individual sensitivity [90]. Regarding spermatogenesis, chemotherapeutic agents may be categorized into low, moderate or high risk. The high-risk category includes alkylating (e.g., cyclophosphamide and ifosfamide) and platinum (e.g., cisplatin) agents [91]. In a large retrospective study, Chow et al. evaluated pregnancy rates in male and female survivors of childhood cancer. A total of 5640 male survivors were included, not exposed to pelvic or cranial radiotherapy. The Authors observed that greater doses of alkylating drugs and cisplatin were significantly associated with a reduced probability of having a pregnancy in comparison with their siblings. However, semen parameters were not considered [92]. Several studies reported reduced paternity rates in childhood cancer survivors [93, 94]**,** but semen analysis was performed only in few cases. In a population of 51 male adults long-term survivors of childhood acute lymphoblastic leukemia, those treated with low-dosage of cyclophosphamide had sperm quality and fertility rates comparable with controls, but the serum free-testosterone was lower, suggesting a long-term impairment of Leydig cell function [95]. In the same way, Green et al. observed a correlation between increasing dosage of alkylating agents and increasing risk for azoospermia and oligozoospermia in 214 adult male survivors of childhood cancer (median age at diagnosis 7,7 years), while age at diagnosis was not correlated [96]. Standard first-line chemotherapy in many patients may be compatible with at least a partial spermatogenesis recovery in the long term, though it is unknown who will successively require treatment intensification [97]. Most studies evaluated only hormonal profile as a marker of fertility. Van Casteren et al. evaluated 248 long-term survivors of childhood cancer and they observed a significantly decreased inhibin B levels and increased FSH levels in men treated for Hodgkin and non-Hodgkin lymphoma, acute-myeloid leukemia, neuroblastoma, and sarcoma as compared to other malignancies. Cumulative dosages of procarbazine and cyclophosphamide were the only independent chemotherapy-related predictors [98]. In 2016, Brignardello et al. showed high prevalence of hypogonadism and high values of serum FSH in a cohort of 199 childhood cancer survivors, suggesting a high risk of gonadal dysfunction in this population [99].

In conclusion, overall data point toward a testicular damage of chemotherapy at any age, in both prepubertal and pubertal age. However, long-term effects are unpredictable, and it is not yet possible to predict which cancer survivors will experience fertility problems. More and more cancer survivors are worrying about their fertility, as long-term life expectancy has improved. For this reason, physicians should always suggest semen cryopreservation to all young patients and their parents, independently from the patients’ age. Actually, cryopreservation is possible in most adolescents [100–102].

## Exogenous causes

### Effects of endocrine disruptors

Endocrine-disrupting chemicals (EDCs) are a wide category of exogenous chemicals or mixtures of compounds that interfere with any aspect of hormone action, causing adverse effect on the health of exposed subjects and/or of their progeny [103]. Their classical mechanism of action involves interference with hormone binding to the corresponding receptor, notably the androgen receptor (AR) or the estrogen receptor (ER) [104, 105]. Male reproductive hormonal axis can be heavily influenced by exposure to EDCs even from the very first stages of fetal life until adulthood [106]. Data from a recent meta-analysis support that single EDC effect is probably lower than expected, whereas the “cocktail” effect arising from the exposure to different chemicals may cause relevant alterations [5]. Data from animal models support a role for EDCs exposure in the alteration of testis function during adolescence [107, 108]. Among EDCs, dioxins and other organochlorine compounds are surely the first and widest distributed compounds with demonstrated testicular disruption [109, 110]. In a peripubertal cohort of 516 subjects with serum evaluation of dioxin levels, sperm DNA methylation was evaluated 10 years later at 18 years old: the authors identified 52 differentially methylated regions associated with lowest and highest serum dioxins concentrations, suggesting that peripubertal environmental exposure is related with sperm DNA methylation in late adolescence/early adulthood [111]. Similarly, a study on 152 young men with pubertal evaluation of dioxin exposure and semen analysis at the age of 18 years showed lower semen volume and progressive motility in the higher quartile of dioxins [112]. These studies focused on pubertal exposure to dioxins and later early adulthood seminal parameters, with no information on the effects during adolescence. Dioxins interference on sex hormones levels was observed in a cohort of adolescent boys with in utero and childhood exposure to dioxins: prenatal DDT levels were associated with LH and testosterone reduction in adolescence, after adjustment for Tanner’s stage [113]. Regarding polychlorinated biphenyls (PCBs), a longitudinal study on 438 adolescents with PCB quantification in utero and at age of 14 years reported lower serum concentrations of both LH and testosterone in the higher prenatal PCB exposure group [114], suggesting an interference on hypothalamic-pituitary function rather than a direct testicular damage.

Bisphenol A (BPA) is another pervasive environmental toxicant with potential negative effects on sperm parameters and hormone levels. In general, in vitro and pre-clinical evidence strongly hints that BPA can adversely impact testis function and negatively regulate spermatogenesis, but clinical evidence is scant and controversial. Furthermore, evidence of reproductive harm during the transition age is virtually absent [115]. To date, only one study has evaluated urinary BPA in 671 boys aged 9–18 years, reporting inverse association between BPA exposure and late progression of testicular development and pubertal onset [116].

Among plasticizers, phthalates and perfluoroalkyl substances (PFAS) have gained increasing attention for their endocrine-disrupting properties. Epidemiological studies reported an association between phthalates exposure and altered seminal parameters or sex hormones [117], but only one study evaluated reproductive function in adolescents. In this paper, higher levels of maternal phthalates were associated with reduced testicular and seminal volume and with increased FSH and LH [118]. In a cross-sectional study on 225 Taiwanese adolescents aged 13–15 years, the Authors reported a negative association of PFAS with testosterone levels and an increase in estradiol in highly-exposed males [119]. Only one study reported reduced sperm count and motility in a small group of highly-exposed young men aged 18 years in comparison with controls [120].

Heavy metals have been reported inducing testicular damage, in particular cadmium [14]. A very recent study on 133 boys aged 15–17 reported that combined exposure to toxic metals was associated with increased testosterone and LH [121]. A positive association of heavy metals with total testosterone levels was also observed in the NHANES cohort of adolescents [122], suggesting a compensatory mechanism possibly due to inefficient recognition of testosterone on its receptor, as confirmed by elevated LH levels. A study on 111 adolescents (age 12–14) with increased urinary cadmium levels reported delayed onset of puberty, reduced testicular volume, but reduced testosterone and LH levels, probably due to a direct toxic effect of cadmium on the Leydig cells [123]. The discordance in the association with testosterone and LH levels could be due to the specific evaluation of cadmium only, compared with the combined exposure to different heavy metals in other studies, which further supports the need for a detailed and comprehensive evaluation of different EDCs classes and compounds in toxicology studies.

### Effects of obesity, fat mass, insulin resistance and DMT2

Childhood obesity and pediatric DMT2 are dramatically increased in latest years [124, 125]. In particular, obesity had been associated with reduced testis function, but it has to be clarified if impaired testis function is a concourse or a consequence of impaired metabolic health. The relationship between pubertal development and body weight in boys is controversial [126]. No association between age at pubertal onset and body composition was found in a study on 179 healthy Danish children [127]. Conversely, in another Danish caseload of 218 obese children the Authors observed an earlier testicular enlargement in obese boys compared to normal-weight [128]. Others had reported the onset and progression of puberty in a significant positive relationship with weight and BMI, though it does not seem to be a linear correlation. In particular, mildly increased BMI (overweight) had been associated with earlier puberty, whereas very high BMI (obesity) had been associated with later puberty [129]. In the same way, in underweight boys a delay at every stage of the development might be observed [130]. However, changes in BMI may primarily reflect changes in height rather than changes in body composition during childhood. Fat percentage may therefore better describe the relationship between obesity and puberty, with delayed puberty reported in both very lean and very obese boys [131]. On the contrary, sexual hormones seems to correlate with BMI. A negative correlation between testosterone levels and BMI had been reported in USA obese adolescents, though with normal values of LH and FSH. Two years after bariatric surgery, testosterone improved and the negative correlation between testosterone level and BMI was confirmed [132]. Taneli et al. observed an impairment of Leydig cells function from Tanner stage 2 in obese adolescents [133]. However, numerous factors should be carefully considered. Nutritional factors, physical activity, socio-economic status, ethnicity are able to influence adiposity development [134] and they may be a possible explanation to discrepant results. In a group of adolescents with metabolic disorders at 17 years, or insulin resistance (IR) at 20 years of age, the authors observed impaired testicular function and altered hormone levels compared to those without metabolic disorders. Non-alcoholic fatty liver disease (NAFLD) at 17 years was associated with an almost 50% reduction in sperm output at 20 years of age, while the presence of IR at 20 years was associated with a 20% reduction in testicular volume [135]. In the same way, Kurku et al. observed diminished testosterone and inhibin B levels in pubertal obese boys with NAFLD, whereas no significant differences were detected according to pubertal status, AMH and testicular volumes [136]. When race is considered, African American and Hispanic boys have a higher prevalence of obesity compared to white boys in USA, but they have different correlation with puberty incoming. Hispanics showed no significant differences in timing of puberty respect to weight while African American showed a trend of late puberty in obese [129]. Different results may be recorded depending on the observed population [137, 138]. Finally, it is important to underline the effect of concomitant insulin resistance on pubertal development, which it’s not completely elucidated. Insulin resistance physiologically begins in early puberty and resolves by the end of puberty in normal weight children. Therefore, adolescents are insulin resistant compared with prepubertal children and adults [139]. However, insulin resistance is an important feature of obesity, consequently puberty should be considered a high risk period for developing obesity-related disease [140]. Regarding testicular function, a positive correlation between testosterone and insulin sensitivity was reported in pubertal boys, with testosterone concentration significantly lower in obese and DMT2 males in comparison with lean males [141]. More recently, Nokoff et al. evaluated reproductive hormones in early puberty (Tanner stages 2 and 3). They found significantly lower levels of total testosterone and SHBG in obese boys, though bioavailable testosterone was not different in comparison with normal-weight boys. Insulin sensitivity was significantly associated with higher SHBG and total testosterone [142]. On the contrary, lower total testosterone, free testosterone and calculated free testosterone were previously reported in young pubertal and post-pubertal obese males (Tanner stage ≥ 4) [143].

To date, most studies point out towards a strict correlation between obesity, insulin resistance and testicular function in pubertal boys. However, this association must be better clarified with large population studies. Choice of the right parameters for obesity and stratification for age and Tanner stage have to be done. In the same way, ethnicity and socio-economic status as well as other important confounding factor should be considered.

### Effects of lifestyle factors

Different unhealthy lifestyle, such as smoking and alcohol consumption, are frequently associated with impaired reproductive health. Although both alcohol and tobacco consumption are a widespread habit in adolescents worldwide, the available scientific literature regarding the effects of these lifestyles on testicular function are mainly limited to middle-aged men. Nonetheless, the pubertal and post-pubertal window represents the most sensitive period for testicular function. Adolescents are more sensitive to alcohol and less tolerant of its detrimental effects compared with adults. For these reasons, adolescence represents a key time for the settlement of strategies of intervention and prevention of unhealthy risk behaviors [144]. Gianfrilli et al. recently reported that smoking (32.6%), drinking (80.6%) and use of illegal drugs (46.5%) are common in Italian adolescence [145]. Consequently, it should be extremely important to analyze eventual endocrine and reproductive alterations. Also, the WHO estimates that adolescent alcohol abuse is increasingly widespread [146]. Alcohol is often considered socially acceptable, but its negative effects on gonadal function have been frequently reported. In the testes, alcohol can adversely affect Leydig and Sertoli cells. In vivo and in vitro studies showed that alcohol abuse impairs also the hypothalamic-pituitary–gonadal axis [147] and it is associated with reduced testosterone levels [148]. Research in humans and animals revealed that early alcohol consumption can result in delayed pubertal development [149]. However, all these associations are poorly studied in young men. In the study of Gianfrilli et al. for the first time they observed an association between alcohol abuse and testicular parameters in adolescents. Alcohol was the factor with the greatest negative impact on testicular volume, with a greater than 4- or 5-mL reduction, respectively in moderate (mean of 2 alcohol units per day during weekends) or heavy (mean of > 3 alcohol units per day during weekends) drinkers [145].

Regarding tobacco smoking, it is a common habit in most countries, but its use in reproductive age could be detrimental for gonadal function, as well as drug abuse [150]. Numerous studies have reported the presence of heavy metals in seminal fluid [151]. In particular, higher concentrations of heavy metals such as lead and cadmium have been directly associated with alterations of seminal parameters, in particular progressive motility and viability and reduced fertilizing capability of sperm cells [152–154]. Among the different sources of heavy metals exposure, cigarette smoking is surely one of the most prominent [155]. Heavy metals are involved in the pathogenesis of testicular disruption mainly by oxidative stress, apoptosis and tight junctions toxicity, ultimately leading to male infertility [13, 14]. Moreover, chronic smoking increases liver metabolism of testosterone, while at the same time inducing secretory dysfunction of Leydig and Sertoli cells [156]. Concerning drugs use, cannabis has been shown to negatively impact male fertility, as cannabinoid receptors are expressed in the anterior pituitary, Leydig cells, Sertoli cells and in testicular tissues [157]. Exposure to cannabidiol, one of the most abundant phytocannabinoids present in the plant Cannabis sativa (marijuana), is associated with a reduction in testis size, number of germ cells and Sertoli cells, fertilization rates, and plasma concentrations of hypothalamic, pituitary and gonadal hormones [158]. Nonetheless, it should be noted that while in vitro evidence suggest that cannabinoids might centrally inhibit testosterone secretion, in vivo evidence is controversial, especially in regards to the link with spermatogenesis [159]. However, knowledge is still limited, and additional research is required to elucidate fully the mechanisms of action, as well as the reversibility of cannabinoids effects on the reproductive system during developmental stages.

### Effects of sexually transmitted diseases

Bacteria, parasites, fungi and viruses can potentially infect all components of the male reproductive system, and consequently interfere with reproductive function. Specifically, sexually transmitted infections (STIs) represent a hot topic, especially in adolescents who deal with the first sexual experiences, often in the absence of an adequate information on the possible risks of unprotected coital relationships. An increase in STIs had been reported until 2012, with a following decline [160]. It is now established that the age of the first coital experience is steadily lowering; in Italy it settles around 15.6 years [161]. In US adolescents and young adults, *Chlamydia trachomatis* and *Neisseria gonorrhoeae* represent the most frequent pathogens [162]. Also, HIV and HPV are very frequent [163]. whereas the prevalence of HSV-2 among people aged 14–19 is 0.8% [164]. *Candida albicans* is also considered rare in pediatrics, but more common in adolescents. Mascarenhas et al. reported a prevalence of infection of about 22% in Brazilian sexually active female adolescents [165]. WHO estimates that about 500 million people acquire a STI each year [166], and it is estimated that teenagers/adolescents account for about 50% of new STIs cases [167]. A recent study showed that students perceive a great risk being infected with HIV/STIs, although pregnancy was seen as a more hazardous consequence of unprotected sex [168].

#### Chlamydia trachomatis

*Chlamydia trachomatis* is the most common STIs, both in the general population and among adolescents. In USA, almost two thirds of reported cases seem to be among adolescents and young adults aged 15 to 24 [162]. It affects females more frequently. In the United States, Chlamydia rates increased from 2013 to 2017 in both male and female adolescents. Specifically, the trend increased faster in males [169]. In males it causes non-gonococcal urethritis, but it may manifest with an orchitis-epididymitis, prostatitis and it may evolve in an obstruction of the genital tract [170]. However, in most cases, it appears to be completely asymptomatic. Some in vitro studies showed that C. Trachomatis is able to interfere with sperm motility [171], though other studies didn’t show significant modifications in exposed semen samples [172]. Several in vivo studies demonstrated no relationship between C. Trachomatis antibodies (semen IgA and serum IgG) and alteration of semen parameters [173, 174].

#### Neisseria gonorrhoeae

*Neisseria gonorrhoeae* infection has declined in recent decades, but still remains the second most frequent STIs in general population and adolescence [167]. It typically causes urethritis, which is only rarely able to spread to other parts of the male reproductive system. In these cases, the pathogenic mechanism that might determine infertility seems to lie in the excessive production of reactive oxygen species (ROS), secreted by activated leukocytes. ROS could damage sperm DNA and plasma and mitochondrial membrane, causing a negative effect on sperm motility and vitality and on the DNA integrity [175].

#### Human immunodeficiency viruses (HIV)

Approximately 1 in 5 new HIV diagnoses occurs among individuals aged 13 to 24 years. By the end of 2016, approximately 51000 adolescents and young adults in the United States were living with HIV, and an estimated 40% were unaware of their HIV serostatus [176]. HIV infection can cause a significant deterioration in the reproductive capacity [177, 178] as well as its treatment [179]. Not infrequently, it may cause a reduction in testosterone levels [180]. Pubertal onset can also be delayed in untreated HIV infection [181], though new treatments may have benefits [182].

#### Human papilloma viruses (HPV)

Genital HPV is very frequent in females of all ages, even children, whereas sexual activity might increase risk for genital high-risk HPV infection [183]. In males, HPV virus is often asymptomatic but impaired sperm motility had been reported [184]. A 2015 paper showed a prevalence of HPV infection between 2 and 31% in men from general population and between 10 and 35.7% in males affected by unexplained infertility. The infection was associated with an impairment of sperm motility and the presence of anti-sperm antibodies [185]. A recent systematic review and meta-analysis confirmed a significant reduction of sperm progressive motility [186]. Another recent study reported the same results, also observing an increment of the sperm DNA fragmentation index [187].

## Conclusions

Male reproductive health might be impaired by numerous factors. Adolescence represents a critical window for gonadal development and consequently it should be considered a key time for intervention and prevention of later andrological diseases. To date, there is an emerging global adverse trend of high-risk and unhealthy behaviors in male adolescents. A significant proportion of young men with unsuspected and undiagnosed andrological disorders engage in behaviors that could impair testicular development and function, with an increased risk for later male infertility and/or hypogonadism during the adult life. For these reasons, more studies and greater attention to the adolescents are surely needed.

## Data Availability

No data or material to share.

## Code availability

No code to share.
